# Environmental challenge trials induce a biofluorescent response in the green sea urchin *Strongylocentrotus droebachiensis*

**DOI:** 10.1038/s41598-024-77648-4

**Published:** 2024-11-04

**Authors:** Thomas Juhasz-Dora, Stein-Kato Lindberg, Philip James, Tor Evensen, Samuel Ortega

**Affiliations:** 1 Bantry Marine Research Station, Bantry, P75 AX07 Ireland; 2 School of Biological, Earth and Environmental Sciences, University College Cork, Cork, T23 N73K Ireland; 3 Nofima AS, 9019 Tromsø, Norway

**Keywords:** Biological fluorescence, Animal physiology

## Abstract

Stress in sea urchins leads to high mortality and economic losses in both the environment and aquaculture. The green sea urchin *Strongylocentrotus droebachiensis* has been documented emitting complex biofluorescence, yet how this responds to external stressors is unknown. Adult sea urchins (*n* = 210) were divided between control (*n* = 30) and experimental groups (*n* = 180), using three transport variables: out of water, in water at elevated temperatures, (warm-water) and in water at seawater temperature (cold-water). Hyperspectral imaging of external fluorescence and fluorospectrometric analysis on coelomic fluid was measured at five intervals (hour 0,3,6,9,12). External green emissions (∼580 nm) responded to all treatments, peaking at h9. External red emissions (∼680–730 nm) in the cold-water remained low until an h9 peak. The warm water increased emissions at each interval, peaking at h9. The out of water gradually increased, with the highest at h12. The coelomic fluid fluorescence (∼680 nm) was low to nonexistent except in warm-water, whose elevated levels suggest that fluorescent emissions are a measurable byproduct of internal adaptation(s) to stress. Early detection of fluorescent emissions (broken spines, lesions) may prevent economic losses. The observed link between fluorescence and the applied stressors provides a baseline for developing non-invasive technology for improving echinoderm welfare.

**Supplementary Information:**

The online version contains supplementary material available at 10.1038/s41598-024-77648-4.

## Introduction

Commercial scale harvest of wild sea urchin stocks is increasingly difficult due to the formation of aggregations of sea urchins known as sea urchin barrens. These sea urchins decimate the macroalgae forests, creating a monoculture of starved sea urchins with commercially worthless roe. Therefore, there is increasing interest to develop a roe enhancement industry which involves the harvest and subsequent aquaculture of sea urchins to increase the size and quality of the roe to produce a high value product for the international seafood market. Sea urchins in intensive aquaculture are exposed to various stressors, including handling^1^, shipping^2^, water temperature fluctuations^3^, and secondary disease outbreaks^4,5^. High mortality and subsequent economic losses occur in aquaculture operations when sea urchins are stressed. For example, aquaculture operations of the Asian sea urchin *Strongylocentrotus intermedius* have regular outbreaks of black mouth disease, leading to significant financial losses^5,6^ Similarly, the desiccation and secondary moisture loss through spawning and esophageal fluid excretion experienced by sea urchins exposed to dry transport is considered a primary cause of mortality^2,7,8^ Minimizing mortality events during live transport of sea urchins is key to the long-term economic stability of sea urchin aquaculture, where premium prices from live transport to markets or processing facilities is needed^9^. As there are currently no effective therapeutants for treating causal agents of morbidity and mortalities in sea urchins^1^, reducing stress should be considered the key preventative measure for increasing the productivity and reliability of echinoderm aquaculture.

Stress is known to suppress echinoderm immune function, leaving the animals vulnerable to secondary viral or bacterial infections^1^. The coelomic fluid of the purple sea urchin *Paracentrotus lividus* was found to be dominated by three freely circulating immune cell types (amoebocytes, vibratile cells, phagocytes)^10–12^. The health of a sea urchin is linked to the homeostasis of red and white amoebocyte numbers^11^. The red amoebocytes, otherwise known as red spherule cells (RSC), carry the anti-bactericidal naphthoquinone red pigment echinochrome A, which is known to rapidly increase in the number in sea urchins experiencing stress inducing conditions^10,11,13,14^. This coelomocyte is considered as a primary immune cell in the coelomic fluid of *S. droebachiensis*^15^. Escalating in density during stress events such as bacterial invasions, RSC degranulate, releasing their bactericidal substances into the coelomic fluid^15–18^. Increases in pigment levels within the coelomic fluid is linked to the cellular apoptosis^19,20^. The elevated levels of apoptosis in sea urchins have been shown to be caused by stressful environmental conditions^19,21^.

*S. droebachienensis* RSC coelomocytes have been documented producing multi-tiered fluorescence^15^. The strong, multi-color auto fluorescence shifts to green fluorescence from orange after a maximum excitation intensity (5–30 min)^15^. The general fluorescence emission of *S. droebachiensis* coelomic fluid of the species was found to be in the red part of the spectrum (∼680 nm)^22^. The external biofluorescence of *S. droebachiensis* is emitted by three different sources: intact spines with a broad low green emission (∼550–600 nm), broken spines and lesions with a dominant peak in the green part of the spectrum (∼580 nm), and a red emission peak (∼680–730 nm) originating from a red exudate produced from pores surrounding the anus^22^. What is not understood from the research conducted to date is whether the biofluorescence produced in *S. droebachiensis* directly correlates to stress inducing events.

The aim of this study is to determine whether the biofluorescence produced by *S. droebachiensis* is responsive to external stressors typically experienced during aquaculture conditions, namely elevated water temperatures and out of water shipping. Furthermore, we investigated whether (1) hyperspectral imaging is an effective method for measuring changes in external fluorescence, (2) fluorospectroscopy is effective for assessing coelomic fluid fluorescence, and (3) whether the complex fluorescence emissions produced in this species vary in their intensity in response to stressors as assessed by these two technologies.

## Results

### External fluorescence

**Fig. 1:**
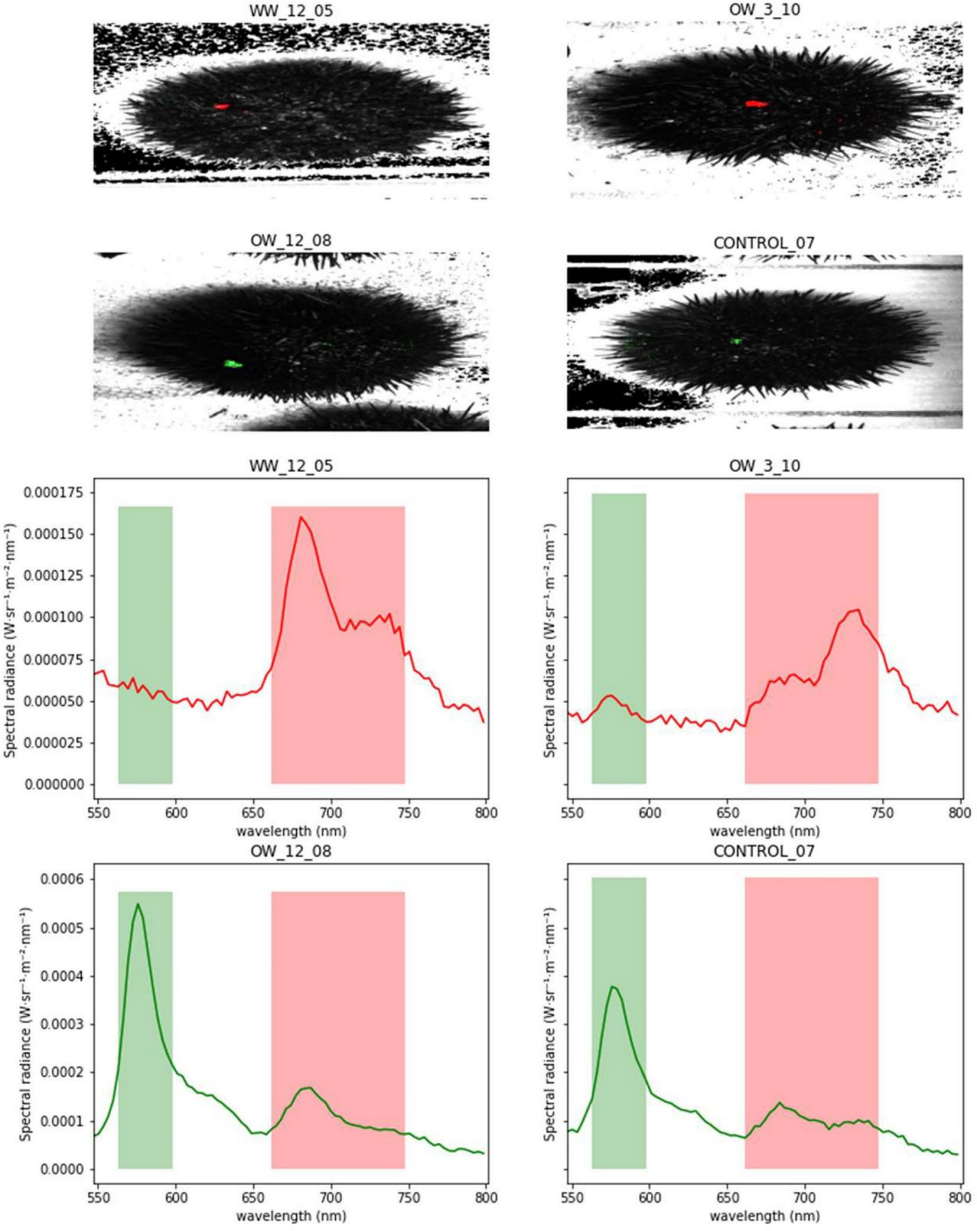
Examples of green (~ 560–600 nm) and red (~ 660–750 nm) fluorescence emissions produced by the green sea urchin *Strongylocentrous droebachiensis* during the environmental challenge trials. The green fluorescence is produced from broken spines and lesions (OW_12_08, CONTROL_07) while red fluorescence is from red exudate (HW_12_05, OW_3_10) produced by exudates from pores surrounding the anus.

The fluorescence spectral analysis of individual urchins revealed distinctive red and green spectra as outliers with high area under the curve (AUC) during all time intervals and experimental variables (see Fig. 1). After identifying these characteristic spectra, it was possible to pinpoint the origin of the emission spectra within the sea urchins’ anatomy. Examples of these regions isolated during hyperspectral analysis of a sea urchin can be observed in Fig. 1. These fluorescent emissions were isolated to broken spines and lesions (green line; ∼560–600 nm), and a red exudate (red line; ∼660–750 nm) produced from the glands surrounding the anus region on the top of the sea urchin. The highest emission produced within the sea urchin spectral data occurs in the green emission peaks produced by broken spines and skin lesions. Each green emission peak was followed by a much shorter peak in the red part of the spectra (∼660–750 nm). Conversely, the emission produced by the red exudate emission intensity reached the lowest levels as those produced by the broken spines and skin lesions. The red exudate produced very subtle to no fluorescent emission peaks in the green part of the spectra. As not all sea urchins exhibited these distinctive fluorescence peaks, these data are valuable for measuring the extent of physical damage or determining the quantity of red exudate produced by an individual sea urchin.

**Fig. 2:**
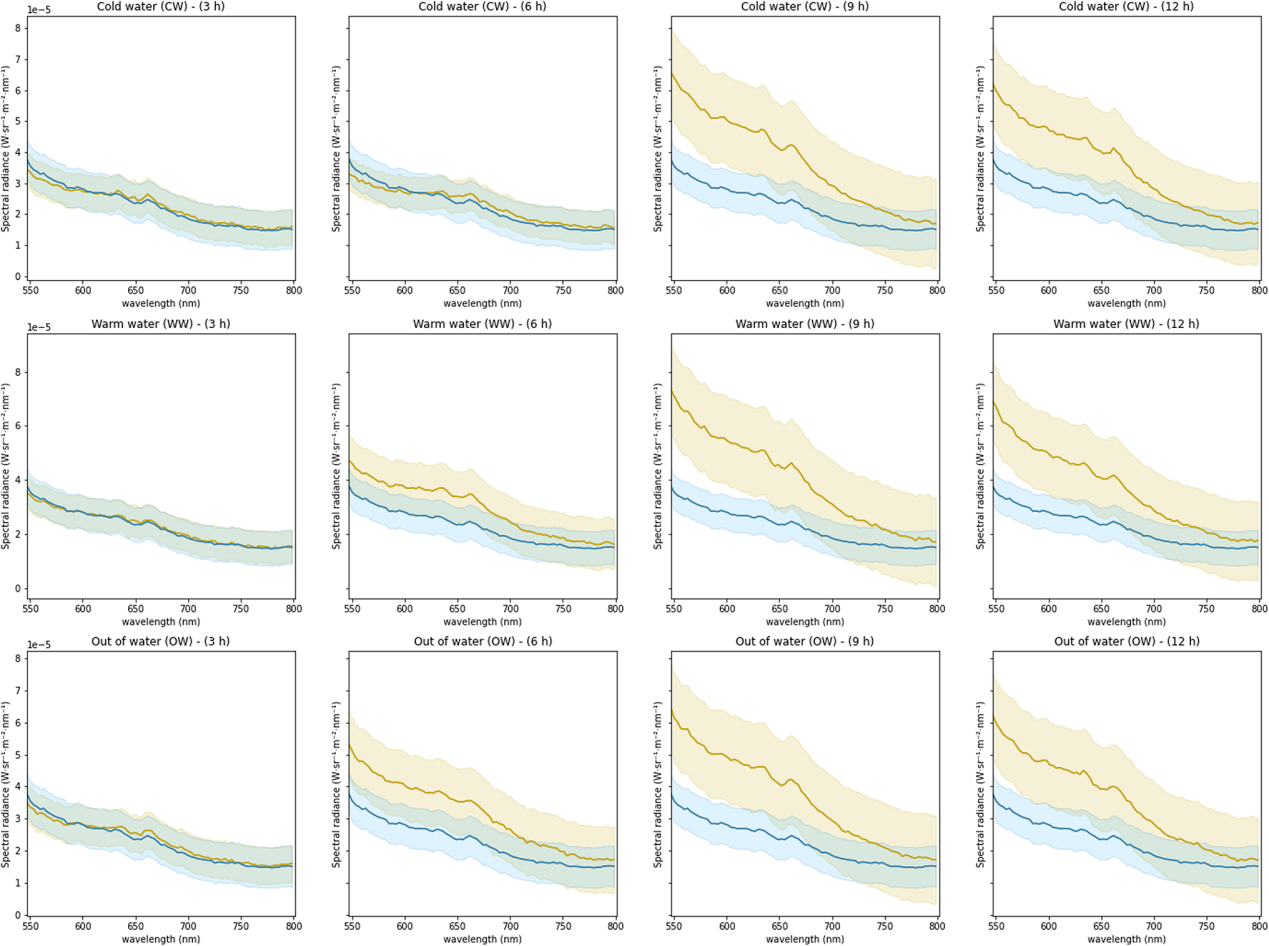
External full spectral range fluorescence emissions (~ 550–800 nm) with the standard deviation (± SD) produced by the green sea urchin *Strongylocentrous droebachiensis* in response to exposure to three environmental variables (cold water, warm water, out of water) as sampled per a 12-hour period. The control group at time zero is denoted with the blue line. The sea urchins within each group increased their fluorescence emissions but varied in their intensity according to sampling time.

Further evaluation of the full spectral range fluorescence (∼550–800 nm) emitted by *S. droebachiensis* detected time-based shifts in intensity that varies in accordance with the environmental stressor applied (see Fig. 2). The full spectral range fluorescence produced by the sea urchins exposed to the three environmental variables (cold water, warm water, out of water) all measured an increasing emission spectra within the experimental timeline (Fig. 2) but varied in their intensity according to individual physiological response as well to sampling time. Increasing emission spectra were observed in the full spectral range spectra from hour 3 to hour 6 in the warm water and out of water groups in Fig. 2, while the cold-water group spectra remained relatively consistent. The emission spectra peaked at hour 9 and remained elevated at hour 12. The standard deviation within the groups is the smallest at hour 3 whilst remaining similar in size in hour 6 only within the cold-water group. The standard deviation increases noticeably at hour 9 in all groups.

**Fig. 3:**
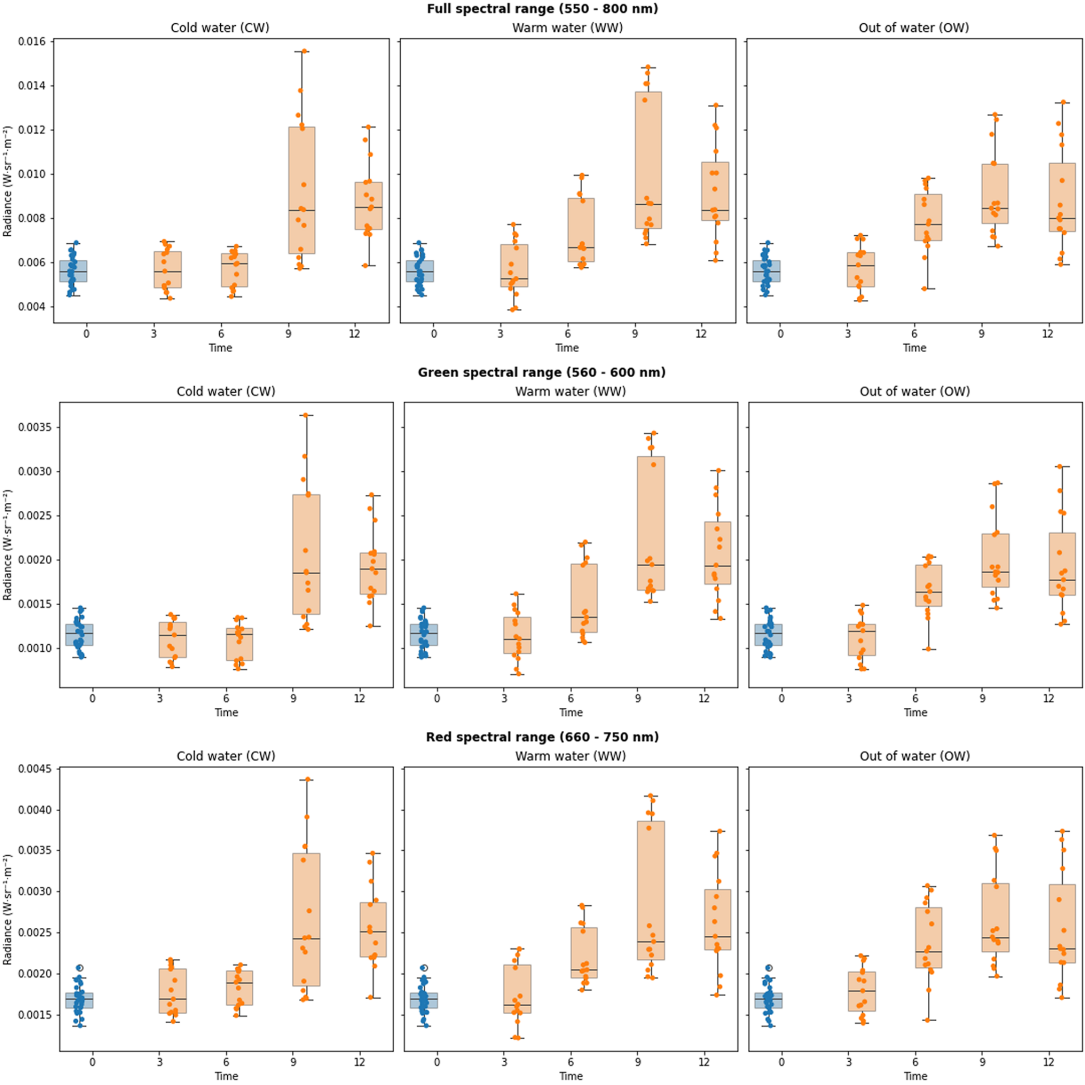
Box plots documenting the three external fluorescence emissions (full spectral range, green, and red) produced by the green sea urchin *Strongylocentrous droebachiensis* in response to exposure to three environmental variables (cold water, warm water, out of water) as sampled every 3 h per a 12-hour period (in orange). The control group (*n* = 30; in blue) from time 0 is added as a reference for each variable group. The sea urchins within each group increased their area under the curve but varied in the intensity of spectra produced by individual sea urchins according to sampling time.

The AUC for the different treatments and the different spectral ranges under analysis are shown on Table 1. Individual variation remained low in the control group as can be seen by the tight clustering seen in all the boxplots shown on Fig. 3. Likewise, the cold-water sea urchins from hours three and six exhibited similar clustering patterns. The AUCs for the full spectral range (Fig. 3, first row) jumped noticeably at hour 9 in the cold-water group, while the warm water and had a less significant transition between hour 6 and 9. The out of water group maintained a steady transition between sampling intervals with similar numbers of outliers. This suggests that the physiological condition of individual animals plays a role in their response to an external stressor. The warm water group AUC had moderate elevation from the control group starting at hour 3. Conversely, the variation of intensity of red spectra emissions increased from hour three for the warm water and out of water groups, with clear upward shifts in the AUC at hour 6 (Fig. 3, third row). The red spectra AUC increased the most at hour 9 in the warm water group, with the hour 12 AUC becoming like the lower AUCs seen in the out of water group at hours 9 and 12. A higher individual variation can be seen occurring at hours 9 and 12. The AUC of cold-water urchins maintained a measured shift in emission intensity hours 3 and 6, with a peak at hour 9. The intensity of emissions declines from hour 9 to hour 12 but remains higher than prior to hour 6. The sea urchins exposed to warm water responded quickly, with increasing emissions observed at each sampling timepoint, reaching the highest emission peak of any environmental variable occurring at hour 9. The out of water sea urchins experienced a steady increase in fluorescence emissions that remained higher at hour 12 than the other two environmental variable groups. The green spectral range AUCs (Fig. 3, second row) reflected only minor variations from the full spectral range and red spectral range.

**Table 1:** The results of the two-way ANOVA analysis of time and treatment of the green spectral range (~ 560–600 nm) external fluorescence spectral emissions produced by the green sea urchin *Strongylocentrotus droebachiensis*.

Variable	DF	Sum sq	Mean sq	F value	*P* value
Time	3	11.423	3.808	59.128	< 2e^− 16^
Treatment	2	0.567	0.283	4.402	0.0137
Interaction	6	1.0310.172	2.667	0.0169	
Residuals	168	10.818	0.064	N/A	N/A

## Coelomic fluid fluorescence

**Fig. 4:**
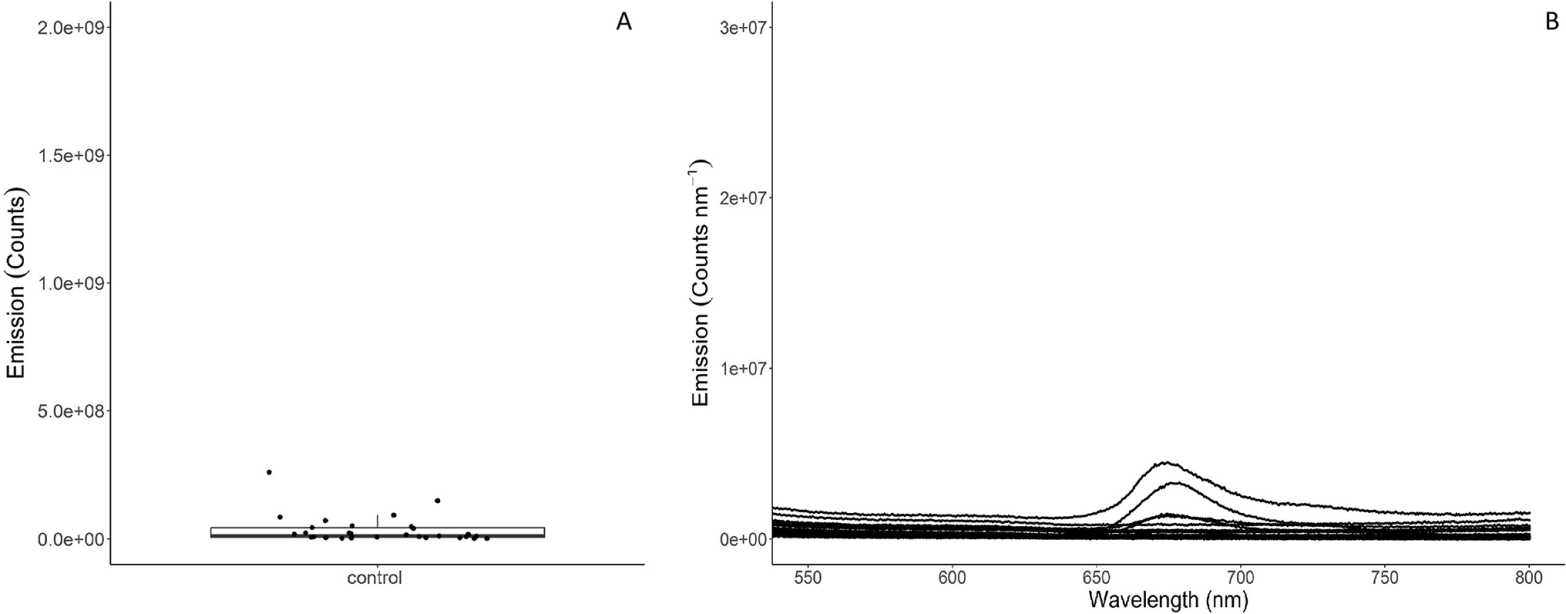
Fluorescence photon counts per nm of green sea urchin *Strongylocentrous droebachiensis* coelomic fluid randomly sampled from animals (*n* = 30) upon arrival at the research facilities (**A**). Fluorescence emittance of the coelomic fluid was overwhelmingly low in the control animals as can be seen by the clustering along the median in the box plots (**B**).

**Fig. 5:**
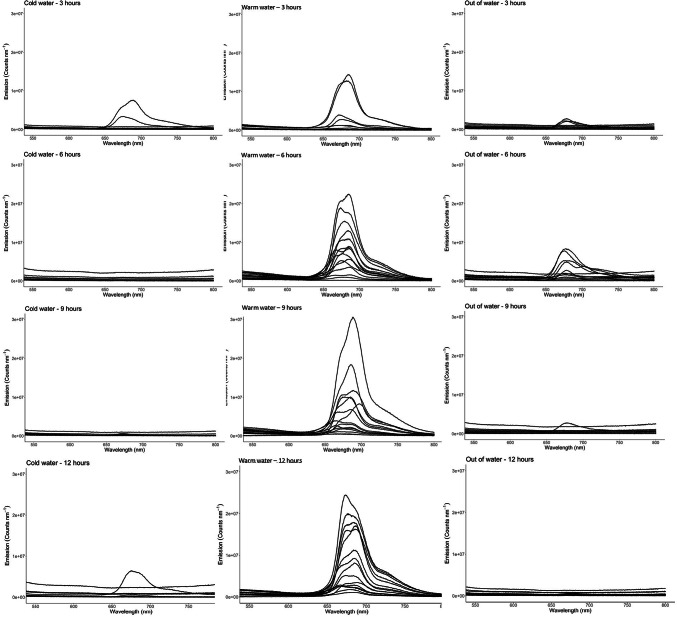
Fluorescence photon counts per nm of coelomic fluid sampled from the green sea urchin *Strongylocentrous droebachiensis* in response to exposure to three external variables (cold water, warm water, out of water) as sampled per 3-hour interval within a 12-hour sampling window. The fluorescence levels in the cold-water replicates remained consistently low (L), while fluorescence levels in the replicates exposed to warm water showed high responses at hour 6,9, and 12 (M). The replicates kept out of water showed an increase in fluorescence at hour 6, while samples from hour 9 and 12 h remained low (R).

**Fig. 6:**
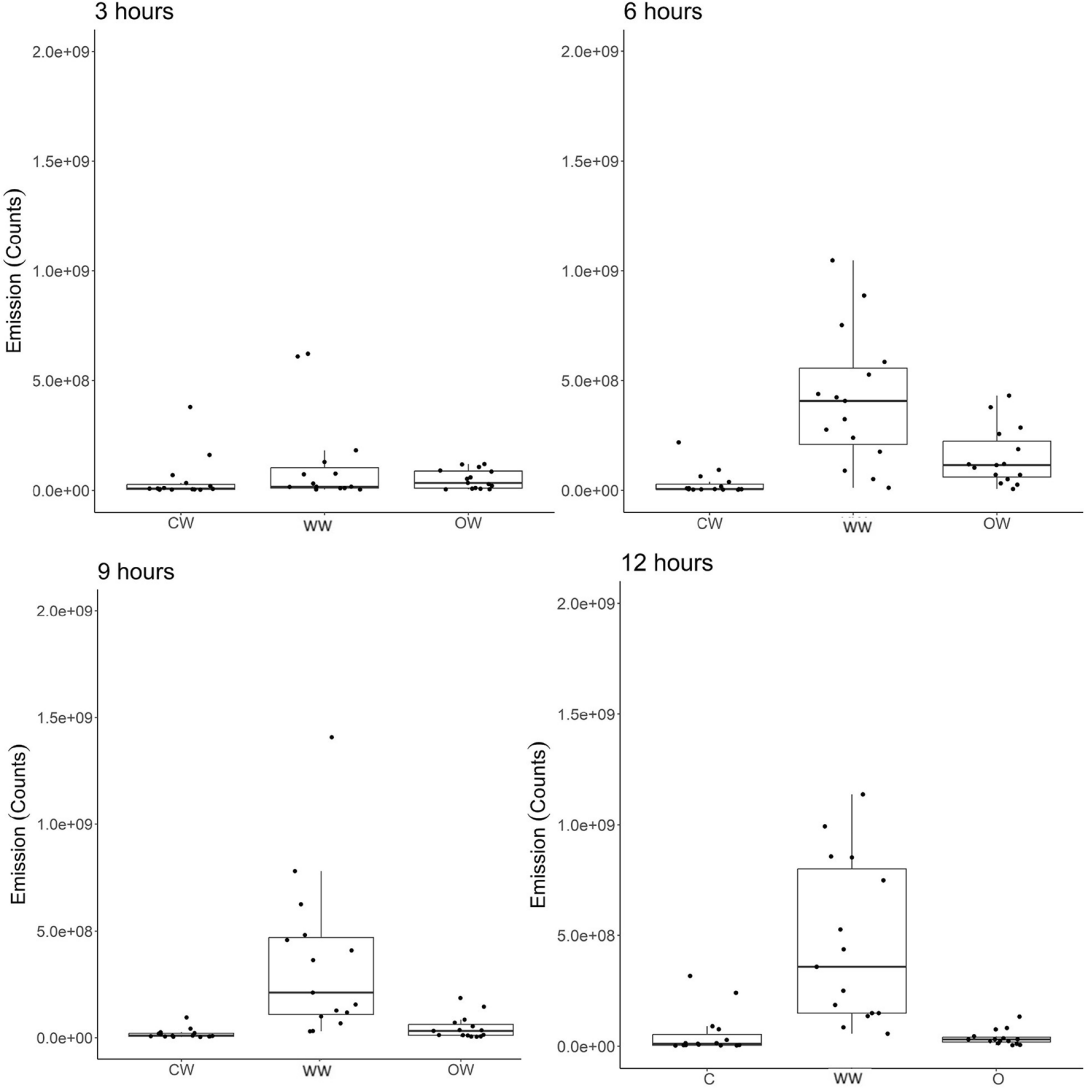
Box plots of the fluorescence count in the spectral range 650–750 nm of coelomic fluid from cold water (CW), warm water (WW), and out of water shipping (OW). The CW and OW analysis detected fewer outliers and greater clustering along the median in comparison to the warm water samples.

The coelomic fluid collected from control sea urchins prior to the commencement of the experiment detected no or low levels of fluorescence (Fig. 4A and B). This is reflected in the tight clustering along the zero-emission line of boxplot (Fig. 4A). This low-level to no fluorescence emission intensity was reflected in the coelomic fluid sampled from the cold-water and out of water sea urchins sampled throughout all time intervals (Fig. 5). There was little variation in the photon counts produced by sea urchins from the cold-water group, as can be seen by the consistent tight clustering in the box plots through time (Fig. 6). However, an increase in individual variation can be observed in the out of water animals at hour 6; this variation decreased to baseline levels in hours 9 and 12.

Conversely, the warm water group already exhibited an elevated coelomic fluorescence at hour 3, with a pronounced density of elevated emissions occurring at hour six and continuing through hour 12 (Fig. 5). Peak emissions occurred at hour 9 in the warm water group. Individual variation occurred through all time intervals, with similar numbers of outliers occurring through all time intervals as seen in Fig. 6. For the warm water treatment group, there was a systematic difference between the tanks. Tank 2 showed a systematically higher response than tank 1 and 3, as well as an increasing trend over time.

## Statistics

We conducted an Analysis of Variance (ANOVA) to evaluate internal (Table 2) and external fluorescence (Tables 1, 3 and 4), using treatment and sampling time as variables. We assessed model fitness through both QQ plots and residual plots available in the Supplementary Material (Figures S3-S6). Correlations between the internal and external fluorescence were observed at 6 h and 12 h for the hot water groups. For cold water and out of water there was no correlation between the fluorescence detected in the coelomic fluid at each sampling interval and that produced by the external anatomy of *S. droebachiensis*.

**Table 2:** The results of the two-way ANOVA analysis of time and treatment of the internal fluorescence spectral emissions produced by the green sea urchin *Strongylocentrotus droebachiensis.*

Variable	DF	Sum sq	Mean sq	F value		*P* value	
Time	3	24.12	8.04	5.495		0.001263	
Treatment	2	178.15	89.07	60.867		< 2e^− 16^	
Interaction	6	39.72	6.62	4.523		0.000281	
Residuals	168	245.86	1.46	N/A		N/A

**Table 3:** The results of the two-way ANOVA analysis of time and treatment of the full spectral range (~ 550–800 nm) external fluorescence spectral emissions produced by the green sea urchin *Strongylocentrotus droebachiensis.*

Variable	DF	Sum sq	Mean sq	F value	*P* value
Time	3	6.821	2.2737	44.345	< 2e^− 16^
Treatment	2	0.287	0.1436	2.802	0.0636
Interaction	6	0.562	0.0936	1.826	0.0967
Residuals	168	8.614	0.0513	N/A	N/A

**Table 4:** The results of the two-way ANOVA analysis of time and treatment of the red spectral range (~ 660–750 nm) external fluorescence spectral emissions produced by the green sea urchin *Strongylocentrotus droebachiensis.*

Variable	DF	Sum sq	Mean sq	F value	*P* value
Time	3	4.634	1.5448	32.179	<2e^− 16^
Treatment	2	0.171	0.0857	1.785	0.171
Interaction	6	0.368	0.0613	1.276	0.271
Residuals	168	8.065	0.0480	N/A	N/A

At 12 h a correlation with the red external fluorescence of − 0.524 was observed with a 95% confidence interval between − 0.817 and − 0.016. The green spectrum and full spectrum measurements showed no significant correlations. At 6 h, the sample correlations were − 0.552 with 95% confidence interval (0.056, 0.83) for total AUC, − 0.560 with 95% CI (0.067, 0.833) for green spectral region AUC, and − 0.537 with 95% CI (0.033, 0.823) for red spectral region AUC.

However, there is a correlation between time and fluorescence emissions within each fluorescence spectra type produced by *S. droebachiensis*. The two-way ANOVA revealed that there was a statistically significant interaction between the effects of time and treatment for internal fluorescence (F (6,168) = 6.62, *p* = 0.000281) as seen in Table 2. A simple main effects analysis on the two variables showed that the sampling time interval in the experiment did have a statistically significant effect on urchin fluorescence (*p* = 0.001), while the environmental variable treatment type also had a statistically significant effect (*p* < 2e^− 16^).

The full spectral range fluorescence has no statistically significant interaction between the effects of time and treatment during a two-way ANOVA analysis (F (6,169) = 1.826, *p* = 0.0967) (see Table 3). However, the sampling time interval did have a statistically significant effect on urchin fluorescence (*p* < 2e^− 16^), while the environmental variable treatment type did not (*p* < 0.0636). On the other hand, the analysis of the relationship between time and treatment for green fluorescence did find a significant difference during a two-way ANOVA analysis (F (6,168) = 2.667, *p* = 0.0169), a significant impact of time (*p* = 2e^− 16^) on urchin fluorescence, and a significant impact of treatment on emission intensity (*p* = 0.0169) (see Table 1). There was no significance in red fluorescence between time and treatment during the two-way ANOVA analysis (F (6,168) = 1.276, *p* = 0.271), a significant impact of time on emission intensity (*p* < 2e^− 16^) while treatment was not (*p* = 0.171) (see Table 4).

## Discussion

Notable knowledge gaps still exist on how the mechanisms used for adaptive stress in echinoderm physiology can be monitored effectively. Several non-invasive assessments have been proposed as welfare indicators to monitor stress in the sea urchin industry, including tube feet adhesion, self-righting behavior, spine responsiveness, and feeding behavior^23^. However, these observations should be considered clinical manifestations of stress which means that physiological adaptations to the stressor(s) have already impacted key functions of sea urchin physiology. For example, it is known that an increase in water temperatures leads to the acidification of the sea urchin coelomic fluid, increasing oxidative stress and gonadal apoptosis^19^. Shifts in the composition and concentration of coelomocytes in the coelomic fluid of echinoderms do occur in response to this oxidative stress^3,11,12^. While these shifts are clear indicators of stress in sea urchins, large-scale monitoring of aquaculture sea urchins with this approach should be considered infeasible. However, the shifting intensity in fluorescent emissions produced by *S. droebachiensis* as documented during exposure to environmental stressors can be considered as a proxy for monitoring the homeostasis of coelomocytes. Hyperspectral imaging can be considered effective for live monitoring method of the red echinochrome pigment levels resulting from the degranulation of coelomocytes in response to stress (red amoebocytes, RSCs). Furthermore, the distinctive fluorescence peaks associated with physical damage such as broken spines and lesions, should also be incorporated within cultured sea urchin monitoring. As such physical insults within sea urchins were not observed during visual inspections within the experimental period, this technique may provide a novel method for refining handling practices as well as serving as an early indicator of infectious pathogens. This method would further enable the quantification of physical damage with high throughput for a large number of individuals. For example, the highly communicable bald sea urchin disease causes conspicuous lesions on the sea urchin body surface due to physical injuries^24^. Likewise, *Vibrio echinoideorum*, a suspected causative agent of sea urchin lesion syndrome, consistently induced lesions within injured sea urchins^25^. As both diseases can cause significant economic losses, fluorescent monitoring of the presence and development of lesions in cultured stock can help limit their financial impact.

Traditionally, hyperspectral imaging technology has been employed to measure the chemical composition of materials being studied, proving to be a valued asset in various fields. These include remote sensing of Earth^26^, forensics^27^, biomedical applications^28^, and food quality inspection^29^, among others. Currently, fluorescence spectral imaging is primarily used for the analysis and characterization of live cells^30^. Recently, it has been discovered that measuring biofluorescence can provide significant insights into marine species. This method has been demonstrated as an efficient and novel approach for evaluating the health and biological condition of diverse set of species, including lumpfish^31,32^, macroalge^33^, and king crab^34^. Fluorescence spectroscopy has similarly been used to assess the post-harvest food quality of marine organisms. For example. the post-mortem variation of freshness (potassium concentrations, pH) in shrimp was tracked through multidimensional fluorescence spectroscopy^35^. Initial veterinary trials have evaluated a spectrometer as a diagnostic tool for studying fluorescent signatures of sampled fluids. By using a luminescence spectrophotometer, fluorescent emissions identified disease-specific profiles of synovial fluid metabolites in domestic dogs suffering from medial compartment disease^36^. The synovial fluids consistently differed from healthy dogs, regardless of breed or sex. Both technologies are well suited for documenting the variability of fluorescent emissions produced by *S. droebachiensis*.

Indeed, S. *droebachiensis* exhibits high phenotypic plasticity in response to environmental factors, making for high levels of intraspecific variation^37–39^ Such variation between individual sea urchins was documented in both the control group as well as with the three environmental variables. There were also differences between the replicate tanks, as illustrated in Supplementary Materials. For the external fluorescence, tank 3 showed higher responses towards the end of the experiment (Figure S1), which could be due to this tank being the last to be sampled. For the coelomic fluid, it is interesting to note that warm water tank 2 showed a steeper increase than warm water tanks 1 and 3 over the course of the experiment (Figure S2). One can hypothesize that tank 2 had a higher temperature, and that the sea urchins are sensitive enough to such a temperature difference that it registers in the coelomic fluid readings.

Nevertheless, measurable increases occurred in the three documented spectra (red, full spectral range, broken spines and lesions) known to be produced by this species. Time was a significant factor in the full spectral range as well as the red and green spectral ranges, which could be due to the animals’ response to the experimental conditions regardless of the treatments. It is not unreasonable to assume that removing the sea urchins from their natural habitat could be a stressor on its own. In the green spectral range both time and treatment, as well as their interaction, turned out significant with a 95% confidence level, while in the red spectral range none of them did. The p values for the full spectral range therefore fall somewhere between the two and would register as significant with a 90% confidence level, but not with 95%. The fluorescence produced by the red exudate therefore seems to be unrelated to the treatment used in this study and instead conditional on other factors. It is interesting to note that the red external fluorescence correlated negatively with the coelomic fluid fluorescence for the hot water group.

If we look at the coelomic fluid fluorescent emissions, sizeable increases in internal fluorescence were occurring in the warm water group. This shift correlated with external fluorescence levels in the warm water 6- and 12-hour groups only. The correlation at 6 h was positive while at 12 h it was negative. which suggests that there is a time lag between the shifts in fluorescent emissions within the animal. However, the confidence intervals for these correlations are quite wide, which makes it impossible to judge the true strength of the correlations given the sample size in this study. On the other hand, the shift from positive to negative correlation from the 6 h to the 12-hour sampling point indicates that the relationship between internal and external fluorescence is more complex than first anticipated. Evaluation of coelomic fluid samples is already used in the related sea stars as a nonlethal diagnostic and research tool for evaluating physiological changes, response to stressors, and disease processes^40^. The scope of coelomic fluid research should be extended to identify the mechanism(s) behind its’ fluorescence and how can be applied to diagnostics currently utilized for echinoderms is warranted.

These increasing fluorescent emissions within the coelomic fluid are likely due to elevated levels of coelomocyte degranulation in response to oxidation stress. Conversely, the fluorescent emissions sampled from the coelomic fluid from the cold water and out of water groups show no to very low responsiveness through the experimental period (see Figs. 5 and 6). This is supported by the negative findings in the Pearson correlation test. The only exception is hour six of the out of water group; this returns to a baseline level in hour nine sampling. The low internal fluorescent response to these experimental stressors may mean that the immune system of the sea urchins has not exceeded their adaptive capacity to the stressor(s). The warm water (18⁰ C) group should be considered the most stressful transport method. This can be seen clearly through the coelomic fluid levels, where the animals were not regulating their fluorescent emissions at any time interval. Though all experimental groups shifted their fluorescence externally, the internal fluorescence levels within the cold and out of water groups remained either low or absent within the sampling periods. These results indicate that temperature during live shipping transport should be considered a key stressor for sea urchins. Heightened temperatures have already impacted commercial long line cultivation of the sea urchin *Strongylocentrotus intermedius*, causing significant declines in production efficiency due to mass mortality events^41,42^. Thus, the increases in fluorescent emissions produced by sea urchins in response to stress could be considered a prelude to clinical stress signs or the onset of mass mortalities within both live shipping and commercial cultivation.

Even though biofluorescence as a health and welfare indicator is novel in the scientific field, this study serves as a precedent for its applicability in a high value aquaculture species. The next step for advancing this technology is to document the baseline biofluorescence levels emitted by this species in typical aquaculture conditions within seawater with minimal to no handling. Once these baseline levels are documented, sea urchins will need to undergo a series of stressor trials to understand the responsiveness of external biofluorescence within these water-based parameters. These experiments should be trialed with the same time intervals, with an increased interval of 15 min within the first two hours on the responsiveness of fluorescence to stressors within their acclimated environment. Further trials should induce experimental infections of sea urchins sustaining mechanical injuries to determine the fluorescent emissions and patterns of early stages of infection of pathogens of concern. This may be specified to a specific bacteria genus due to their unique fluorescent signatures. As echinoderms do not have a well-developed therapeutic index for addressing health issues encountered in aquaculture, developing a methodology for identifying their pre-clinical stress will improve both their welfare and productivity in intensive production. The link observed between *S. droebachiensis* fluorescence and the applied stressors as well as the fluorescent emissions from mechanical damage (broken spines, lesions) not observed during visual inspection, provide a baseline for developing a novel technological method to non-invasively monitor echinoderm welfare. The feasibility of such an application should also be investigated in other invertebrates of economic and ecological importance.

## Methods

The experiments were conducted at Nofima laboratories (Tromsø, Norway) in December 2022. This study contains experimental procedures on non-cephalopod invertebrates performed in compliance with Norwegian national guidelines based on the European Union Directive 2010/63/EU for the protection of animals used for scientific purposes. The green sea urchin *S. droebachiensis* is not of conservation concern nor has any restrictions associated with their use in research. A control group (*n* = 30) was randomly sampled at hour zero of the experiment after being acclimated within the aerated transport tank for 3 h post collection in 3 separate collection bags. The experimental groups were randomly placed into aerated 60-liter tank to immediately begin the experiment. The animals were not fed during the experimental timeline. The randomly assigned experimental groups (*n* = 180) were subsequently conducted in using three environmental variables: out of water shipping transport (3 tanks; *n* = 20 each), in water transport at elevated water temperatures (3 tanks; *n* = 20 each; warm water: 18⁰ C) and in water transport at seawater temperature (3 tanks; *n* = 20 each; cold water: 8⁰C). Two of these environmental variables are known stressors to sea urchins, namely out of water shipping transport^2,9^, and elevated water temperatures (+ 10⁰ C)^3,19,43^ A total of 15 sea urchins (5 per environmental variable tank) were sampled at each experimental sampling interval (3,6,9,12 h). These time point selections are based on equally spaced time intervals within the average shipping time it takes live sea urchins to simulate the transport time between harvesting and reaching aquaculture facilities (~ 12 h). The multiple time points were affirmed by earlier proof of concept trials with the sea urchins to establish the methodology for this study. The details of the experimental sampling period are presented in Table 5.

**Table 5:** Experimental outline of the environmental variables used for stress trials on adult green sea urchins *Strongylocentrotus droebachiensis*. Each environmental variable trial was conducted in triplicate.

Experimental group	Sample time (h)					Total /tank	Total/3 tanks
	0	3	6	9	12		
CONTROL	30	--	--	--	--	10	30
OUT OF WATER*	--	5	5	5	5	20	60
WARM WATER (18⁰ C) *	--	5	5	5	5	20	60
COLD WATER (8⁰C) *	--	5	5	5	5	20	60
TOTAL SEA URCHINS							210

*Triplicate tanks were initially stocked with 20 animals each for each experimental group. Five animals were sampled from a tank at each time interval.

### Sea urchins

**Fig. 7:**
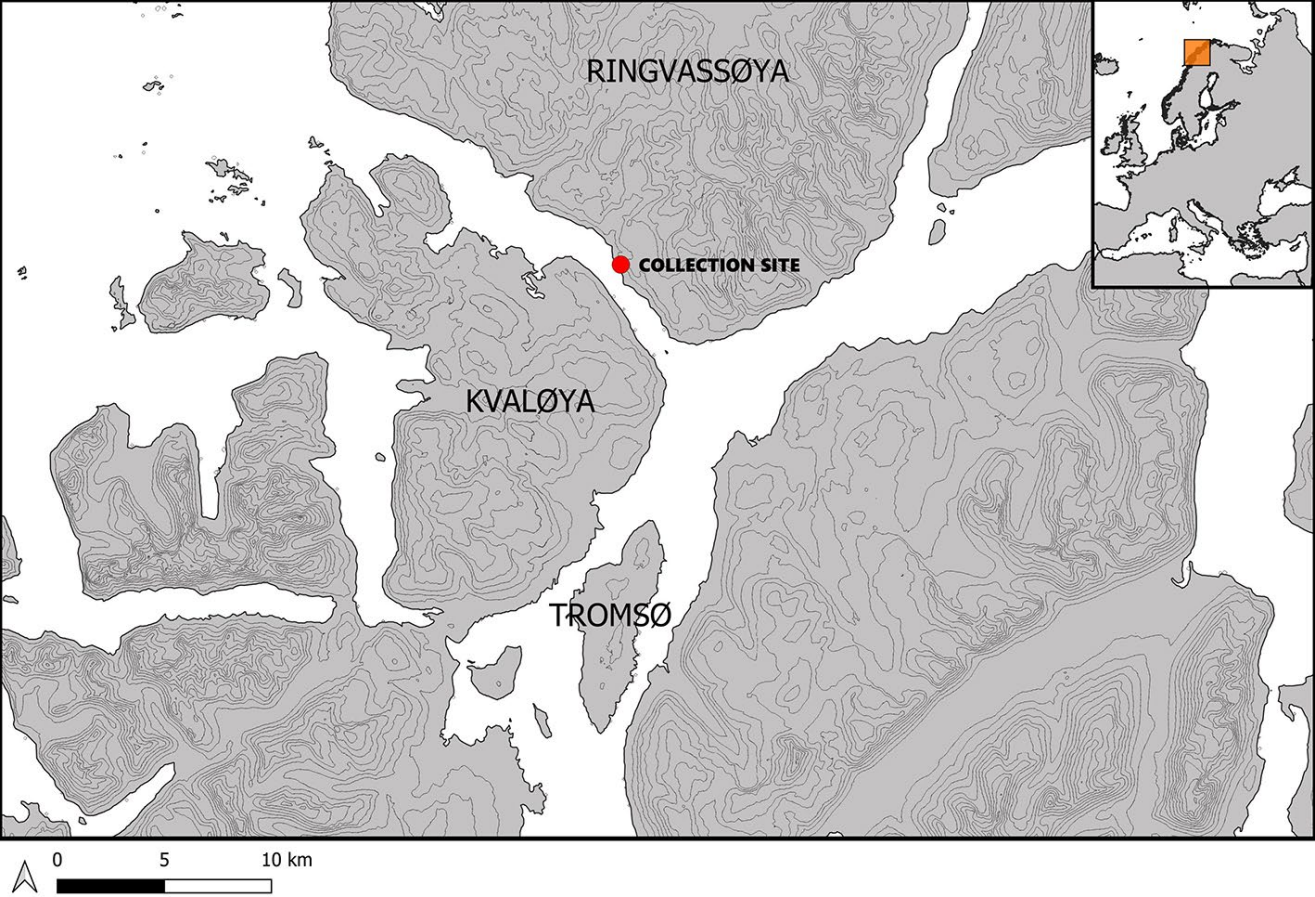
Green sea urchins *Strongylocentrous droebachiensis* were collected from the Kvalsund strait of Northern Norway.

The collection of adult green sea urchins occurred in Kvalsund, Norway, (69° 45′ 16.5312′ N, 19° 2′ 2.0904” E) on 13.12.2022 (Fig. 7). The urchins were collected into mesh bags that were then placed in an ambient temperature seawater filled container (1,000 L) for transport to the Nofima laboratory facilities 5 km away. The sea urchins were acclimated for 3 h post collection prior to being randomly selected into experimental groups. No mortalities of sea urchins occurred during the experimental trial.

## Hyperspectral imaging and Fluorospectroscopy

### Hyperspectral imaging

The hyperspectral imaging of the green sea urchins was conducted with a custom imaging platform consisting of a VNIR-1800 hyperspectral camera (Norsk Elektro Optikk AS, Oslo, Norway) mounted 1 m above resulting above an illuminated conveyor belt with Royal Blue excitation wavelength (~ 445 nm) setting of the G5 XR30 Pro Radion LED reef lighting system (Ecotech Marine Bethlehem, PA USA) for a field of view of 300 mm. The VNIR-1800 hyperspectral camera captures spectral information by the push-broom method, where each frame of captured spectral data consists of a thin spatial line across the field of view (x-axis). The collection of data in the second spatial dimension (y-axis) is conducted by spatially scanning each sample. The working distance between the camera and the samples was 100 cm, resulting in a FOV of 30.6 cm across the conveyor belt (x-axis), with a spatial resolution along the conveyor track in the x direction of 0.17 mm. The sea urchins were placed in groups of 10 on a white Styrofoam tray placed on the conveyor belt for spatial scanning. The capture for the hyperspectral camera ranges in the Visible and Near Infrared (VNIR) spectra (400–1000 nm), 1800 spatial pixels, and a spectral resolution of 5.5 nm. A high exposure time was configured for the hyperspectral measurements (7.52 m/s) to counter the weak fluorescence signals documented during prior analysis. The spatial resolution in the y-axis on push-broom cameras depends on the exposure time and the speed of the conveyor belt. The speed of the conveyor belt was configured to 6 cm/s resulting in a spatial resolution of 0.45 mm in the y-axis.

**Fig. 8:**
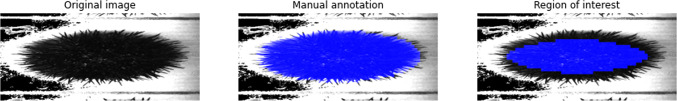
Annotation methodology in the hyperspectral images to analyze the external fluorescence emissions produced by the green sea urchin *Strongylocentrous droebachiensis* in response to exposure to three environmental variables (cold water, warm water, out of water). The manual annotation area and then region of interest is noted in blue. The region of interest was detected by reducing the manual annotation by 20%.

Given the absence of a standard procedure for calibrating fluorescence spectral data, the raw hyperspectral data were radiometrically calibrated using the HySpex Rad v2.5 software (Norsk Elektro Optikk, AS, Oslo, Norway) to produce spectral radiance values in Wm^− 2^sr^− 1^nm^− 1^, as conducted in previous studies^22,32^. All sea urchins were individually annotated from the radiance calibrated images and then cropped into individual images using the Breeze hyperspectral imaging software (Prediktera, Umeå, Sweden). An example of the manual annotation methodology can be observed in Fig. 8, where the manual annotation area and subsequent region of interest is noted in blue.

To prevent potential errors in annotations during the analysis, 20% of the edges of the manual annotations were eliminated using the erosion image morphological operation. The fluorescence spectral data (~ 550 to 800 nm) was extracted from the pixels that correspond to the region of interest (Fig. 2.c) for further analysis. The fluorescence spectral data was further cleaned using the Interquartile Range (IQR) method^44^. The spectral data was analyzed qualitatively using spectral radiance, and the area under the curve (AUC) of the fluorescence spectra was used to quantify the fluorescence radiance recorded in individual animals. Feature extraction and representation from the hyperspectral data was performed using Python (v3.8.10, python.org).

### Fluorospectroscopy

The coelomic fluid (2 ml) was extracted from the peristomal membrane of each sea urchin with a 26-gauge hypodermic needle attached to a sterile syringe (BD microlance, Eysins, Switzerland). As coelomic fluid sampling is considered a stressor and would impact the post sampling fluorescent emissions, sea urchins were humanely euthanised post sampling. Pre-chilled citrate/EDTA anticoagulant (0.5 ml) was preloaded in each syringe based on a formula used for crustaceans [0.45 M NaCl, 0.1 M glucose, 30 mM sodium citrate, 26 mM citric acid, 10 mM EDTA; pH 5.4]^45^. Sampled coelomic fluid was briefly shaken and then placed on crushed ice prior to analysis. The samples were inserted into a 3.5 ml fluorescence quartz cuvette (Science Outlet Inc., Weifang, China) for fluorescence analysis with a Duetta fluorescence and absorbance spectrometer (HORIBA Scientific, Kyoto, Japan). This instrument consists of an enclosed black compartment in the middle of which the quartz cuvette containing the liquid sample is placed. The cuvette is illuminated with the excitation light source from one direction, and the emitted fluorescence is recorded by a sensor placed perpendicularly to the excitation light source. This geometry ensures that most of the recorded signal originates from the sample. Ideally the sample liquid should be transparent to avoid scattering of the excitation light and reabsorption of the emitted fluorescence.

The methodology used for analyzing coelomic fluid fluorescence is synonymous with that used in Juhasz-Dora et al., 2024. EZ Spec Software (HORIBA Scientific, Kyoto, Japan) was used to collect raw data which was subsequently analyzed with R studio^46^. The EZ Spec Software was set to produce excitation light with peak wavelengths at 5 nm intervals between 250 and 350 nm while emitted light wavelengths were recorded from 400 to 800 nm with a 0.5 nm spectral resolution. The integration time was set to 1 s. The emitted wavelength data at 400 nm was selected for further analysis as it yielded the strongest emission signal. The instrument records emittance spectra in radiometric units of counts per µÅ, which were converted to counts per nm. The strongest fluorescence signal was observed between 650 and 750 nm, and the photon count in this range was calculated.

### Statistical analysis

An analysis of variance (ANOVA) test was done for both internal and external fluorescence measurement with treatment and sampling time as the predictors. An interaction term was included since it is reasonable to assume that the response over time depends on the treatment.

For the internal fluorescence, the logarithm of the photon counts between 650 and 750 nm as the response variable. Likewise, the logarithm of radiance was used for the external fluorescence measurements in the wavelength ranges 550–800 nm, 560–600 nm and 660–750 nm. The fit of the models was assessed using QQ plots and residual plots.

A Pearson correlation test was subsequently used to assess whether a relationship exists between external fluorescence emissions and those produced within the coelomic fluid.

## Electronic supplementary material

Below is the link to the electronic supplementary material.

Supplementary Material 1

## Data Availability

The data that support the findings of this study are available on request from the corresponding author.
