# Supplementary material for: Environmental challenge trials induce a biofluorescent response in the green sea urchin Strongylocentrotus droebachiensis
Source: Sci Rep. 2024 Nov 4;14:26671. doi: 10.1038/s41598-024-77648-4 (PMC11535239; doi:10.1038/s41598-024-77648-4)
Supplement: Supplementary file 1 — Supplementary Material 1 [file 41598_2024_77648_MOESM1_ESM.docx]

**Supplemental material: Environmental challenge trials induce a biofluorescent response in the green sea urchin *Strongylocentrotus droebachiensis*.**


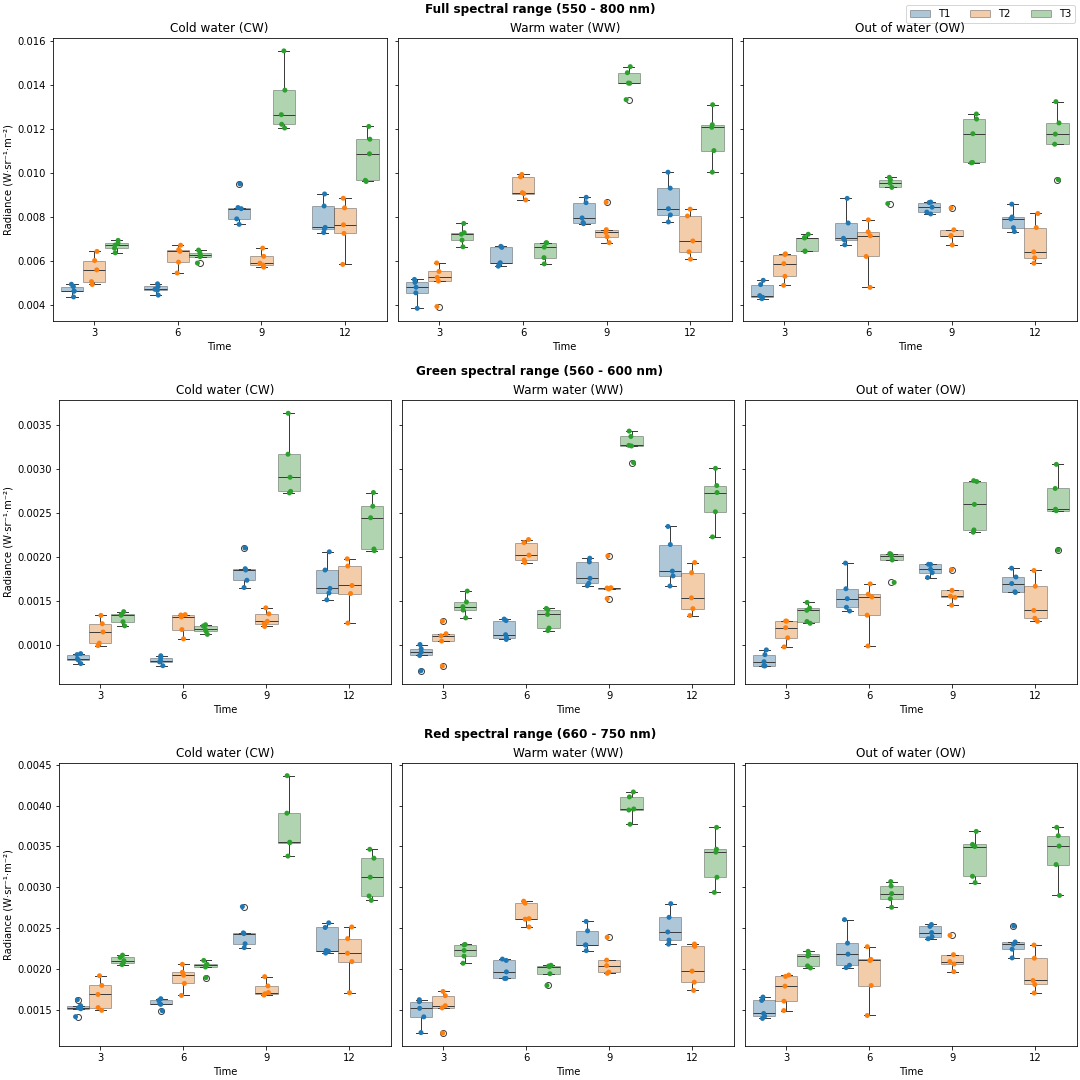


Figure S-1. A box plot depicting the results of the application of three environmental transport variables [out of water], in water at elevated temperatures, [warm water] and in water at seawater temperature [cold water]) on green sea urchins *Strongylocentrotus droebachiensis* (n=150) using the external fluorescence measured at five intervals (hour 3,6,9,12). This refers to data presented in Table 2 of the manuscript.


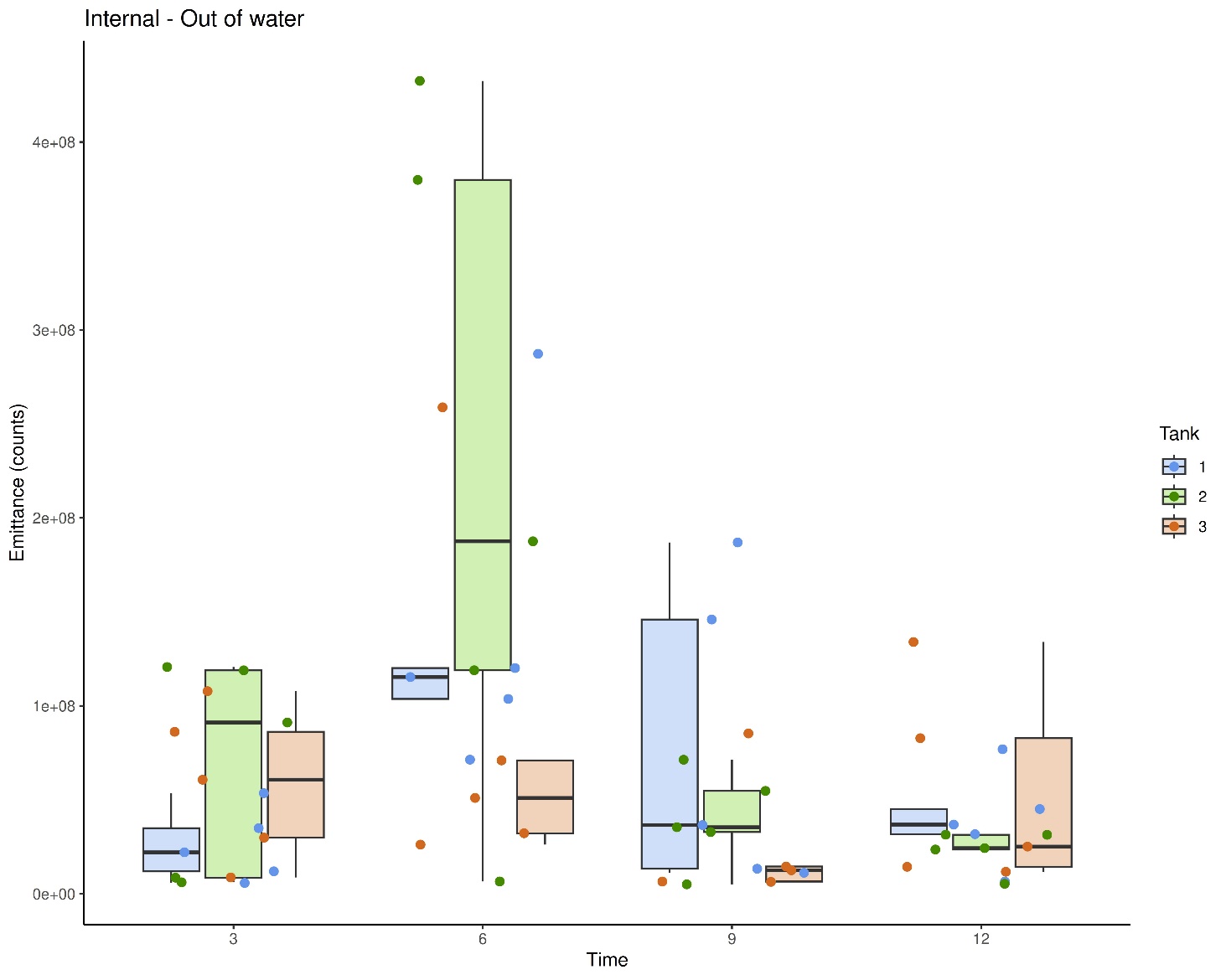


Figure S-2. A box plot depicting the results of the application of the out of water environmental transport variable on the internal fluorescent spectra (~660–750 nm) emitted from the coelomic fluid green sea urchins *Strongylocentrotus droebachiensis* coelomic fluid (n=150), using the external fluorescence measured at five intervals (hour 3,6,9,12). This refers to data presented in Table 2 of the manuscript.


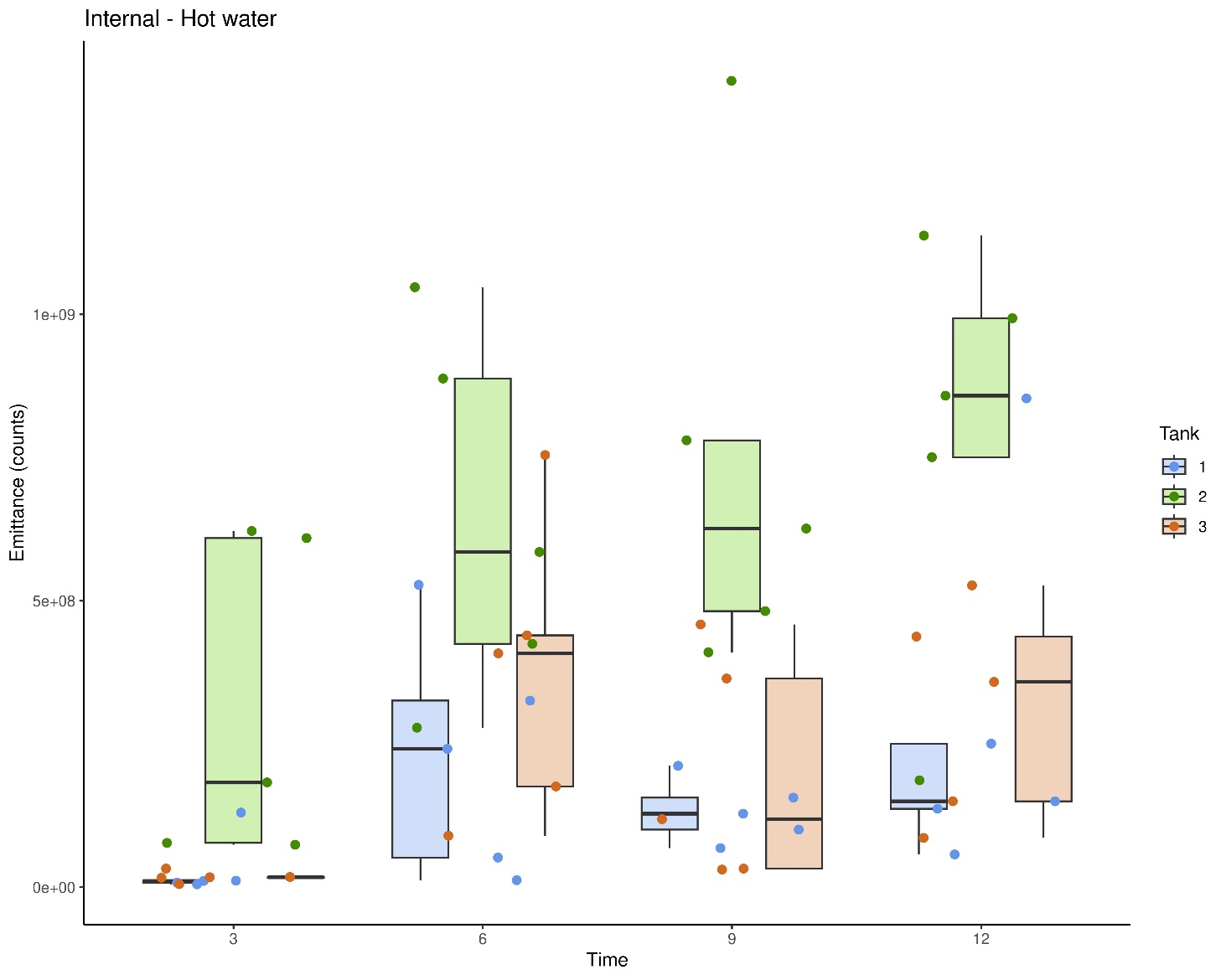


Warm water

Figure S-3. A box plot depicting the results of the application of the warm water environmental transport variable on the internal fluorescent spectra (~660–750 nm) emitted from the coelomic fluid green sea urchins *Strongylocentrotus droebachiensis* coelomic fluid (n=150), using the external fluorescence measured at five intervals (hour 3,6,9,12). This refers to data presented in Table 2 of the manuscript.


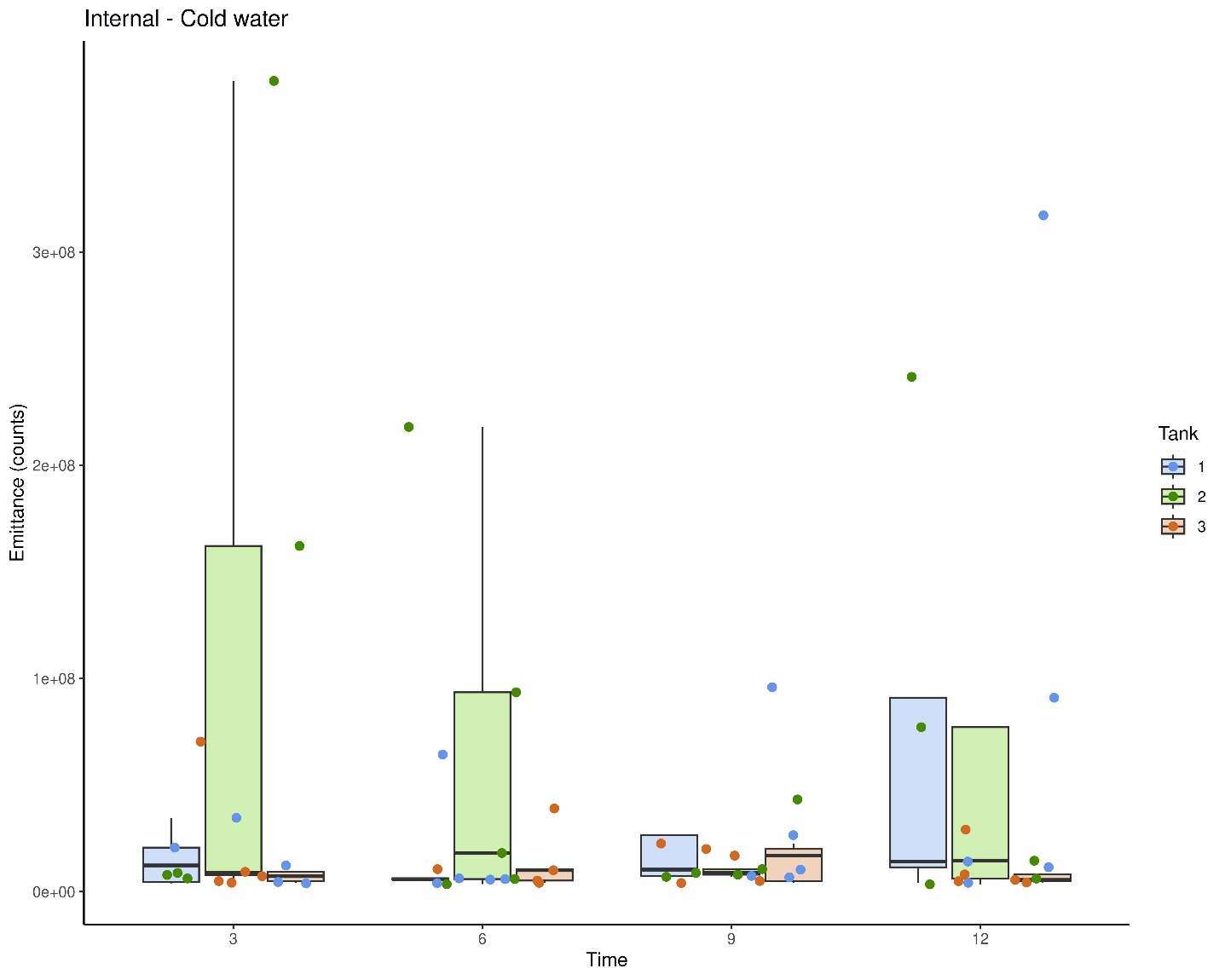


Figure S-4. A box plot depicting the results of the application of the cold water environmental transport variable on the internal fluorescent spectra (~660–750 nm) emitted from the coelomic fluid green sea urchins *Strongylocentrotus droebachiensis* coelomic fluid (n=150), using the external fluorescence measured at five intervals (hour 3,6,9,12). This refers to data presented in Table 2 of the manuscript.


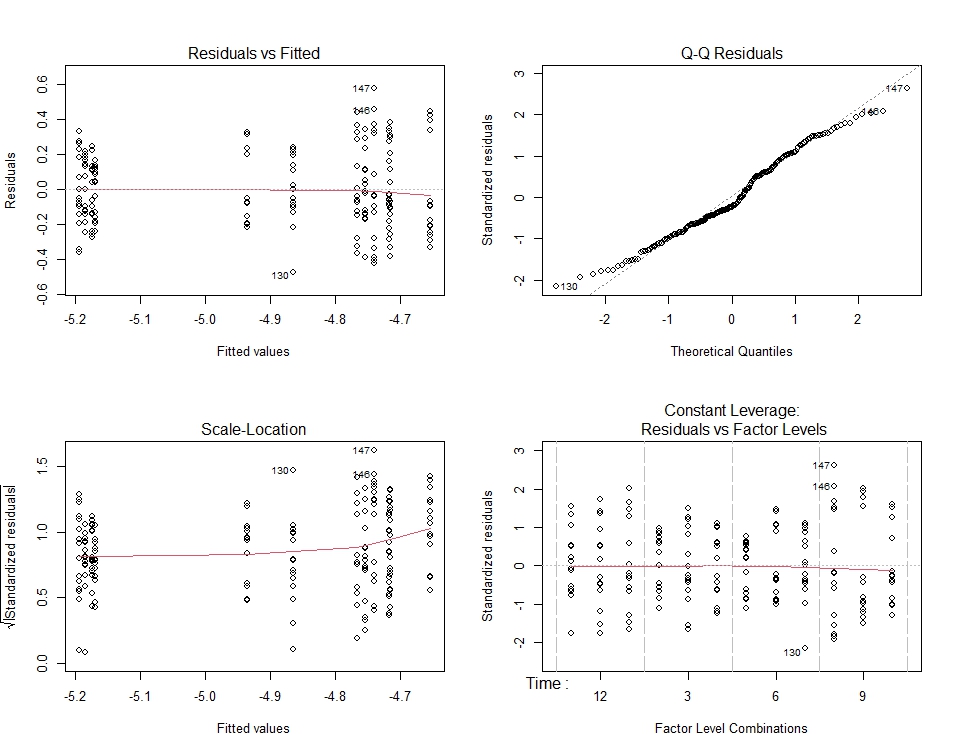


Figure S-5. Diagnostic plots for two-way ANOVA analysis of external fluorescence residuals of all external fluorescent emissions from *Strongylocentrotus droebachiensi* across the full spectral range (500–800 nm). This refers to data presented in Table 2 of the manuscript.


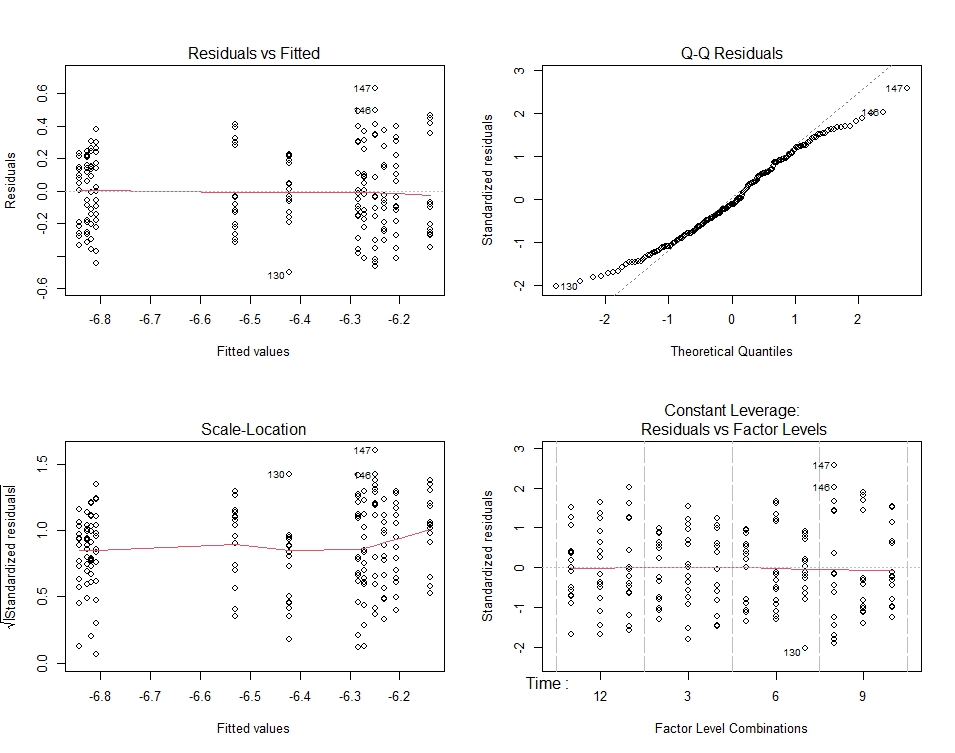


Figure S-6. Diagnostic plots for two-way ANOVA analysis of external fluorescence residuals of all external fluorescent emissions from *Strongylocentrotus droebachiensi* across the green spectral range (660–750 nm). This refers to data presented in Table 3 of the manuscript.


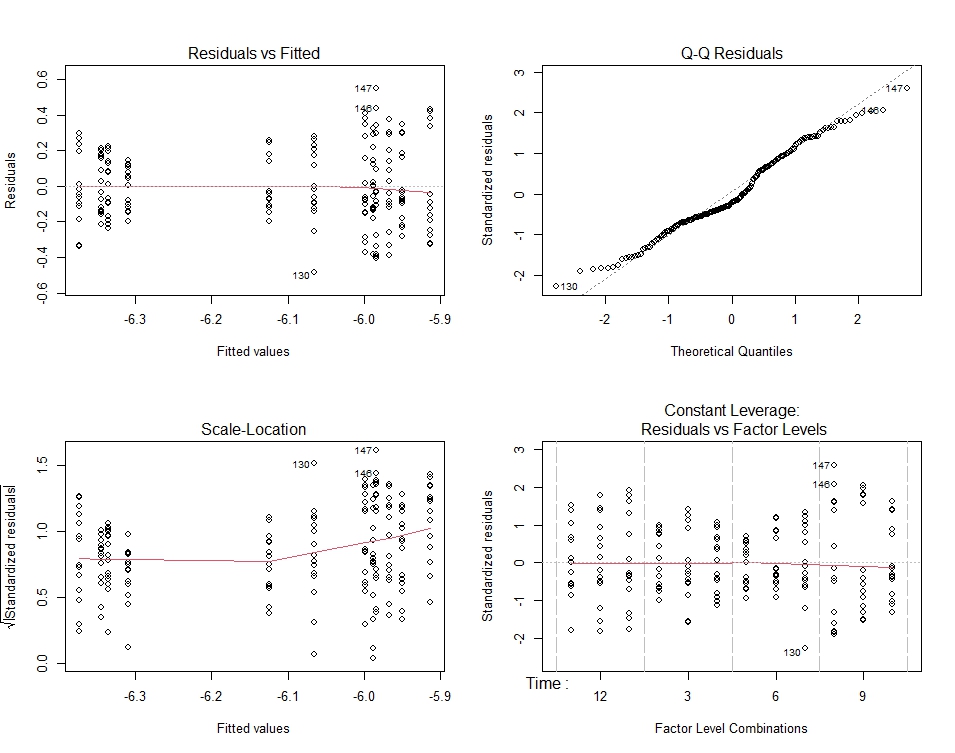


Figure S-7. Diagnostic plots for two-way ANOVA analysis of external fluorescence residuals of all external fluorescent emissions from *Strongylocentrotus droebachiensis* across the red spectral range (660–750 nm). This refers to data presented in Table 4 of the manuscript.


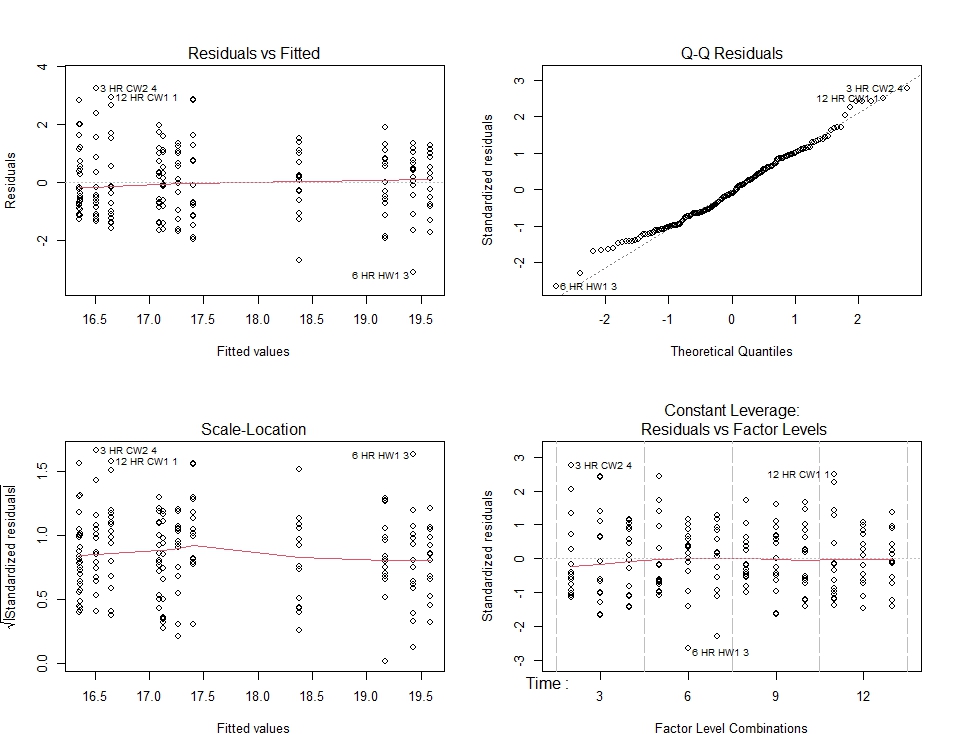


Figure S-8. Diagnostic plots for two-way ANOVA analysis of external fluorescence residuals of all internal fluorescent emissions from *Strongylocentrotus droebachiensi* coelomic fluid across the red spectral range (650–750 nm). This refers to data presented in Table 1 of the manuscript.
